# The influence of international medical electives on career preference for primary care and rural practice

**DOI:** 10.1186/s12909-015-0483-2

**Published:** 2015-11-11

**Authors:** Iain R. Law, Lucie Walters

**Affiliations:** Flinders School of Medicine, Flinders University, Adelaide, South Australia Australia

**Keywords:** International medical elective, Graduate preferences, Rural practice, General practice, Medical education, Curriculum

## Abstract

**Background:**

Previous studies have demonstrated a correlation between medical students who undertake international medical electives (IMEs) in resource poor settings and their reported career preference for primary care in underserved areas such as rural practice. This study examines whether a similar correlation exists in the Australian medical school context.

**Methods:**

Data was extracted from the Medical Schools Outcomes Database (MSOD) of Australian medical students that completed commencing student and exit questionnaires between 2006 and 2011. Student responses were categorized according to preferred training program and preferred region of practice at commencement. The reported preferences at exit of students completing IMEs in low and middle income countries (LMIC) were compared to those completing electives in high income countries (HIC).

**Results:**

The effect of elective experience for students expressing a preference for primary care at commencement was non-significant, with 40.32 % of LMIC and 42.11 % of HIC students maintaining a preference for primary care. Similarly there were no significant changes following LMIC electives for students expressing a preference for specialist training at commencement with 11.81 % of LMIC and 10.23 % of HIC students preferring primary care at exit. The effect of elective experience for students expressing a preference for rural practice at commencement was non-significant, with 41.51 % of LMIC and 49.09 % of HIC students preferring rural practice at exit. Similarly there were no significant changes following LMIC electives for students expressing a preference for urban practice at commencement, with 7.84 % of LMIC and 6.70 % of HIC students preferring rural practice at exit.

**Conclusions:**

This study did not demonstrate an association between elective experience in resource poor settings and a preference for primary care or rural practice. This suggests that the previously observed correlation between LMIC electives and interest in primary care in disadvantaged communities is likely dependent on student and elective program characteristics and supports the need for further research and critical examination of elective programs at Australian medical schools.

## Background

Student interest in international medical electives (IMEs) has prompted researchers to evaluate their educational potential and role in medical curricula [1]. A recent literature review suggested that IME experience in resource poor settings is associated with a preference for primary care and work with underserved populations [2].

The best evidence for this association is published in several US-based single institution comparative studies. For example, Ramsey and colleagues found that 36 % of doctors participating in an IME program in a low income country as medical students chose general practice compared to 11 % of controls [3]. Godkin and Savageau found that students undertaking IMEs were more likely than their colleagues to express interest in primary care and working with underserved populations [4]. Similarly, compared to non-travelling colleagues, trainee doctors completing IMEs during training were more likely to work with underserved populations and at risk groups; work in rural areas; have a public health degree; and to switch from a sub-specialty into general medicine [5, 6].

Despite including suitable control groups, caution must be exercised when interpreting the results of these studies as: (i) none used longitudinal data to evaluate changes in career preferences before and after elective experiences; (ii) all of the programs included applicant selection processes that likely generated a significant selection bias; and (iii) these studies focus on individual, well structured, and supported elective programs. As acknowledged by all authors, these limitations make it impossible to infer causality or generalize beyond the participating students and doctors.

This study responds to these limitations and evaluates the transferability of previous findings to the Australian context. Specifically, we analysed questionnaire data collected by the Medical School Outcomes Database (MSOD). These questionnaires are offered to all students enrolled in Australian medical schools and are administered at commencement of studies and at graduation. Consequently, this study provides a novel analysis of career preference before and after elective experience using a large and representative sample.

## Methods

### Data source

MSOD is the principal data collection project for medical student information in Australia, implemented and operated by the Medical Deans of Australia and New Zealand to evaluate educational programs and inform workforce planning. Questionnaire data are collected directly from medical students from all 18 medical schools in Australia [7]. Student elective data are supplied to MSOD directly from each medical school and reconciled with questionnaire responses. Prior analysis of these data indicates that 50 % of graduate entry students and 35 % of high school entry students undertake IMEs, with more than half completing at least one elective in a resource poor setting [8].

Data from MSOD were provided to the authors in May 2013, and included elective data and selected questionnaire data for students completing both the Commencing Medical Student Questionnaire (CMSQ) and Exit Questionnaire (EQ) during the period, 2006–2011 [9, 10]. This range provides the maximal number of responses available in the dataset at the time of analysis. Considering that the duration of medical school in Australia ranges from four to six years and eligible respondents had to have completed both the CMSQ and EQ, only the 2006–2008 CMSQs and the 2009–2011 EQs were included in the analysis. The response rate for the CMSQ was 82 % (2044 respondents), 90 % (2697 respondents), and 95 % (3235 respondents) for the years 2006, 2007, and 2008, respectively [11–13]. The retention rate for the 2011 EQ was estimated at 81 % [14]. Retention rates for the 2009 and 2010 EQs have not been published. Given the limited period for which data was available, and that high school entry courses are typically two years longer than graduate entry programs, it should be noted that graduate entry students are over-represented in this analysis.

### Participants

Only students responding to either question of interest (see *Measures* below) in both the CMSQ and EQ were included in the analyses (*n* = 3673). We assumed that international students completing electives in their country of birth likely had systematically different motivations for undertaking IMEs and were excluded from the analysis, resulting in a total study cohort of *n* = 3596.

Students were categorized as having completed at least one overseas elective in a low or middle income country (LMIC) setting, or as having completed electives only in high income country (HIC) settings, which included electives completed in Australia or overseas. Host countries were categorized as LMIC if they reported a gross national income (GNI) less than US$12 275 in the World Development Index [15]. Due to small numbers of respondents undertaking electives in low income countries (*n* = 212), low and middle income country electives were not stratified.

### Measures

Two streams of analysis were conducted to examine, i) the association between elective experience and preferred training program at EQ taking into account CMSQ preference and, ii) the association between elective experience and preferred region of practice.

#### Preferred training program

The CMSQ and EQ asked students to select from a range of responses for preferred training program. Preferences were categorized into “primary care” when respondents indicated a preference for general practice, public health medicine, or rural and remote medicine, or “specialist”, for all other preferences. Only students that provided answers for the preferred training program question at CMSQ and EQ were included (*n* = 2213). Students reporting their decision as not final at EQ were categorized according to the first of their top three preferences. Respondents’ preference at CMSQ was treated as a confounder and respondents were categorized accordingly, resulting in CMSQ preference categories for primary care (*n* = 309), specialist (*n* = 1508), and undecided (*n* = 396). The preferences reported at EQ for students that completed LMIC electives were compared to those that completed HIC electives. The association was tested using Chi Square.

#### Preferred region of practice

There were no questions in the CMSQ or EQ that asked specifically about work with underserved populations. Rural practice was accepted as a surrogate because rural populations are one of the largest and most visibly underserved populations in Australia. The questionnaires asked students to select from a range of responses for preferred region of practice. Preferences were categorized as “rural” if respondents indicated preference for a regional city, large town, small community, and smaller town or “urban” if they indicated preference for a capital city or major urban centre. Only students that provided answers for the preferred region of practice question at CMSQ and EQ were included (*n* = 2894). Again, respondents’ preference at CMSQ was treated as a confounder and CMSQ preference categories were created for rural (*n* = 656) and urban (*n* = 2238). The preferences reported at EQ for students that completed LMIC electives were compared to those that completed HIC electives for both categories. The association was tested using Chi Square.

### Ethics approval

Ethics approval was provided by all participating Universities for the MSOD Project, and approval for the study was granted by the MSOD Research and Scientific Advisory Committee (SA-2012-013).

## Results

The results for preferred training program can be found in Table 1. There was no significant difference between LMIC and HIC respondents in preference for primary care at EQ for any of the CMSQ preference categories, including primary care (*χ*^2^ = 0.065, df = 1, *p* = 0.80), specialty (*χ*^2^ = 0.732, df = 1, *p* = 0.39), and undecided (*χ*^2^ = 0.510, df = 1, *p* = 0.47).

**Table 1:** Change in preferred training program between CMSQ and EQ

Preference at CMSQ	Elective experience	Preference at EQ		Total (*n* = 2213)
		Primary care	Specialty	
Primary care	LMIC	25 (40.32 %)	37 (59.68 %)	62
	HIC	104 (42.11 %)	143 (57.89 %)	247
Specialist	LMIC	43 (11.81 %)	321 (88.19 %)	364
	HIC	117 (10.23 %)	1027 (89.77 %)	1144
Undecided	LMIC	17 (17.00 %)	83 (83.00 %)	100
	HIC	60 (20.27 %)	236 (79.73 %)	296

The number and proportion of students that expressed an interest in either primary care, specialty training, or were undecided at CMSQ, who completed electives in either HIC or LMIC settings and their subsequent preferred training program at EQ. Percentages in parentheses express each number as a proportion of students in each elective group for each CMSQ preference

The results for preferred region of practice can be found in Table 2. There was no significant difference between LMIC and HIC respondents in preference for rural practice at EQ in either of the CMSQ categories, including rural (*χ*^2^ = 2.781, df = 1, *p* = 0.10) and urban (*χ*^2^ = 0.885, df = 1, *p* = 0.35).

**Table 2:** Change in preferred region of practice between CMSQ and EQ

Preference at CMSQ	Elective experience	Preference at EQ		Total (*n* = 2894)
		Rural	Urban	
Rural	LMIC	66 (41.51 %)	93 (58.49 %)	159
	HIC	244 (49.09 %)	253 (50.91 %)	497
Urban	LMIC	48 (7.84 %)	564 (92.16 %)	612
	HIC	109 (6.70 %)	1517 (93.30 %)	1626

The number and proportion of students that expressed an interest in either rural or urban practice at CMSQ, who completed electives in either HIC or LMIC settings and their subsequent preferred region of practice at EQ. Percentages in parentheses express each number as a proportion of total students in each elective group for each CMSQ preference

## Discussion

This study evaluated data provided by MSOD for a relationship between elective experience in resource poor settings and student career preferences for primary care and work with underserved populations. This analysis did not detect any association between elective experience and career preferences.

The lack of a statistically significant correlation in these data must be interpreted carefully, as failing to find a significant correlation is not the same as demonstrating that it does not exist [16]. However, these findings provide an opportunity to compare and contrast the conditions under which Australian medical students undertake electives and the conditions reported in previous studies. Two aspects in particular may help explain this disparity in results; namely student characteristics and the nature the elective programs.

First, previous studies used selection criteria that likely recruited participants with systematically distinct motivations and career intentions that may have caused selection bias. For example, the elective program studied by Godkin and Savageau required students to submit a written application and essay [4]. The International Health Fellowship Program studied by Ramsey and colleagues considered applications from across the United States and made selections based on their “commitment to international, cross-cultural, or community-oriented primary health care” (p.567) [17]. Gupta and colleagues acknowledged that many trainees chose their site for training based on the opportunity to participate in the IME program [6].

In contrast, the MSOD data were collected from students from across Australia, many of whom arranged electives independently. It is reasonable to infer that this population has diverse motivations for completing electives in LMIC settings. A recent literature review provides insight into what motivates medical students to undertake IMEs. It identified three dominant motivations: (i) altruism; (ii) self-serving rationale, such as language acquisition, exposure to range of illnesses, or access to high patient volumes; and (iii) the opportunity to work beyond the scope of practice typically allowed for medical students [18]. Electives that meet these expectations likely provide considerable learning experience to participating students in terms of clinical competence, confidence, procedural skills, and leadership [1, 19]. However, they also carry risk for students and patients, especially if students are pushed to perform beyond their knowledge and ability with variable quality and availability of supervision [20, 21]. Indeed, such students are at risk of engaging in ‘medical tourism’ , which describes students who visit resource poor settings, but are underprepared, lacking structured educational objectives, and are unable to offer benefit to the host institution [22]. At the time of writing, there are no published studies describing the motivations or experiences of Australian medical students undertaking IMEs in resource poor settings. The present study reinforces the need for further research in the interest of student wellbeing and the welfare of host communities.

Second, the present findings may reflect differences in the electives themselves. Elective programs studied previously tended to include student support and structured learning outcomes. For example, participants in the study reported by Ramsey and colleagues were required to complete a two week full-time course prior to departure and were expected to participate in community health activities as well as clinical placements [3, 17]. Also, several studies make reference to desirable learning outcomes such as cultural competency, international understanding, and development of values of social responsibility achieved through immersion in culturally diverse settings [4, 6, 17].

In contrast, Australian students often organize electives independently and receive limited and variable support and structure. In a recent study, 12 of the 16 medical schools interviewed in Australia reported that they offered pre-departure training, but only half of those programs were mandatory and had an average duration less than five hours [8].

The contribution of these two key differences to understanding the disparity between past and present findings is offered tentatively and subject to confirmation through further research. Subject to this limitation, these findings suggest that the previously established association between elective experience and career preference may be limited to well-structured and supported elective programs that accept students with specific motivations.

It is worth noting that the students and programs previously studied aspire to the recommendations for safe and educational elective programs described in the literature [23–25]. Specifically, these programs tended to select students that had reflected on their motivations for undertaking an IME, provided them with suitable pre-departure training, and supported them throughout the elective to maximize learning opportunities.

While Australian medical students are likely to benefit from the electives that they undertake, the differences in program characteristics invite the question; does this disparity in results reflect substandard structure and support for Australian medical students undertaking electives in resource poor settings to the detriment of student wellbeing, learning, and professional growth? At the very least, these findings establish the need for further research into ways to minimize risk and maximize learning and hopefully encourage medical educators to critically examine the elective programs for which they are responsible.

There are important limitations to this study. First, due to limitations inherent to secondary analysis of data, the analysis employed national GNI as a relatively crude indicator of resource poor elective setting. Inevitably there is considerable within- and between-country variation in terms of healthcare resource allocation. Visiting a LMIC does not guarantee exposure to the kind of resource poor settings previously studied.

Second, there is a large discrepancy between respondent attrition from CMSQ to EQ for speciality of practice (*n* = 1383) compared to location of practice (*n* = 702). This finding is consistent with survey results published on the MSOD website, however the reason for the consistently poorer response rate to the specialty of practice question is unknown [14, 26].

Finally, the parallel between interest in work with underserved populations and rural practice is made tentatively. Despite the fact that rural populations remain relatively underserved in Australia, what constitutes underserved populations in previous studies may be very different. It is possible that if asked directly, LMIC elective students would indicate a greater preference for work with the underserved.

## Conclusions

In conclusion, the results do not appear to support generalization of previous findings that elective experience in resource poor settings correlates with future practice in primary care with underserved populations. To the contrary, this correlation appears to depend on student and elective program characteristics. Further research is required to optimise elective programs in Australia. This study demonstrates that further research should focus on; i) the motivations and characteristics of Australian medical students to undertake IMEs; and ii) how to best structure electives and support students to maximise student learning and safety, while maintaining the integrity of host community healthcare.
